# Comparing Chinese and Western classroom learning environment research: a bibliometric analysis and visualization

**DOI:** 10.3389/fpsyg.2023.1213397

**Published:** 2023-08-24

**Authors:** Juan Cai, Free De Backer, Geert Vandermeersche, Koen Lombaerts

**Affiliations:** Department of Educational Sciences, Vrije Universiteit Brussel, Brussels, Belgium

**Keywords:** classroom learning environment, bibliometric analysis, pedagogical aspect, social and psychological aspect, structural equation modeling

## Abstract

A comprehensive cross-national understanding of the classroom learning environment (CLE) is critical to advancing CLE’s development. This study compares the English and Chinese CLE literature to enhance a complete understanding of CLE. We conducted bibliometric analysis on 3,130 English literature from Social Sciences Citation Index (SSCI) and 1,261 Chinese literature from the Chinese Social Sciences Citation Index (CSSCI). The results show that both Chinese and English literature focus on the influence of CLE on students’ cognitive and affective learning outcomes and the incorporation of information technology in CLE. The results also reveal the differences between Chinese and English literature from two perspectives: (1) Chinese CLE research mainly focuses on English education, and English CLE research mainly focuses on science education and (2) Chinese CLE research mainly analyzed the pedagogical aspect of CLE, while English CLE research focused on CLE’s social and psychological aspects. We also discussed that future research should focus on (1) the balance of analyzing CLE from the perspective of students and other educational stakeholders such as teachers and school leaders, (2) student differences from the pedagogical level, (3) the physical level of CLE, (4) the use of statistical methods such as structural equation modeling that can analyze the mechanisms underlying the effects of CLE on student learning, and (5) the interdisciplinary research on CLE.

## Introduction

The classroom learning environment (CLE) is an important learning space for students (Fraser, 2001; Fan and Dong, 2005), and the quality of the CLE directly affects student learning and development, such as student learning motivation and self-regulation (e.g., Velayutham and Aldridge, 2012). Given that CLE has such a pivotal role in students’ learning, the CLE has received a great deal of attention from researchers in the fields of education, psychology, and sociology (Fraser et al., 2010).

CLE refers to “the social, psychological, and pedagogical contexts in which learning occurs and affects student achievement and attitudes” (Fraser, 1998, p. 3). In addition, Fan and Dong (2005) included the physical perspective of the factors mentioned above, which influence the development, quality, and result of teaching activities. The physical aspect refers to the educational material foundation and the conditions for teaching and learning. The social element relates mainly to the interaction between teachers and students and between students and their peers. The psychological aspect refers to teachers’ and students’ personalities, mental states, and psychological climate. The pedagogical element refers to the instructional methods and assessment.

Regarding CLE’s social, psychological, pedagogical, and physical aspects, the existing studies have been identified and explored to some extent. However, these studies tend to examine these four aspects either separately or in limited combinations, neglecting a comprehensive view (e.g., Aldridge et al., 2012; Chen et al., 2017; Cai et al., 2022a, b). The approach in previous research fails to consider the holistic and interrelated nature of the CLE dimensions. This constitutes a research gap.

This study aims to fill the identified research gap by acknowledging the multifaceted nature of the CLE, which encompasses social, psychological, pedagogical, and physical dimensions. A holistic understanding of these dimensions is not only instrumental in enhancing understanding of the interconnectedness of these aspects but also in how their interplay shapes the overall learning environment. This holistic approach will also allow us to provide a more robust framework for researchers and educators to understand and enhance the CLE (Fan and Dong, 2005), thereby creating a positive CLE for students (Rusticus et al., 2023).

Moreover, while these CLE dimensions are universally applicable, their specific characteristics can vary greatly depending on the cultural context (Aldridge and Fraser, 2000). The impact of these cultural contexts on CLE is a very important topic. Pioneering work by Cai et al. (2022a, b) and Dorman and Adams (2004) highlighted that characteristics of their CLE could vary significantly based on their cultural backgrounds. For example, the Chinese CLE, traditionally shaped by its historical and societal parameters, tends to prioritize exam-oriented content (Lau and Lee, 2008), a competitive environment, teacher dominance (Wang C. et al., 2018), and larger class sizes (e.g., Ning et al., 2015; Guo et al., 2022). In contrast, the Western CLE usually embodies a more supportive assessment protocol, a cooperative class atmosphere, student-led initiatives, and smaller classes (Lee et al., 2009). This necessitates a comparison study of Chinese versus Western CLE, which will offer unique insights into classrooms in different cultural contexts and can point out the potential learning opportunities for different countries to optimize their own CLE (Fraser et al., 2010). Most importantly, a comprehensive comparison of China’s CLE with multiple Western contexts would provide a more nuanced understanding of cultural influences on CLE, thereby fostering a more equitable and inclusive CLE (Abacioglu et al., 2023).

However, current research predominantly focuses on two-way comparisons, such as those between China and America (Jia et al., 2009) or China and Australia (Aldridge and Fraser, 2000). Their approaches, though valuable, restrict the scope of understanding the global dynamics of CLEs.

Hence, this study endeavors to address the evolution of CLE over time, focusing on Chinese versus Western CLE through a bibliometric approach. A bibliometric approach is essentially a mathematical and statistical method. It interprets scientific knowledge by analyzing bibliographic data in a deductive and visually appealing manner (Börner et al., 2003). Unlike traditional review methods, a bibliometric approach can handle a larger volume of articles, leading to a more objective analysis of the Chinese and international research fields in CLE (Chen, 2016). It outlines key themes, and developmental trends, and bridges the existing gap in cross-national studies.

In this context, we have employed CiteSpace, a bibliometric visualization software. Developed by Professor Chaomei Chen of Drexel University’s School of Information Science and Technology, CiteSpace leverages the Java computer programming language to facilitate our research (Chen, 2016).

The data for this analysis was gathered from both the Chinese Social Sciences Citation Index (CSSCI) and the Social Sciences Citation Index (SSCI). Most Chinese social scientists tend to publish their higher-quality work in journals from the Chinese Social Sciences Citation Index (CSSCI). In contrast, the Social Sciences Citation Index (SSCI) features only a few of their works (Shu et al., 2020). Additionally, social science studies like CLE research are more locally and regionally focused than natural science (Gong and Cheng, 2022). Thus, combining CSSCI and SSCI is an effective strategy to reveal a comprehensive picture and compare the characteristics of Chinese and international social science research (Shu et al., 2020; Gong and Cheng, 2022).

Against this background, this article aims to address the following two research questions:

1. What are the characteristics, hotspots, cutting-edge, and knowledge base between the literature in Chinese and English?
2. What research gaps can be addressed in future CLE research?

## Methods

### Literature search procedure

This paper retrieved the data on November 12, 2022 and extracted English literature from the Web of Science (WoS) core collection in the SSCI and Chinese literature from the CSSCI database. The same keywords (classroom learning environment*) OR (classroom psycho* learning environment) OR (classroom pedagog* learning environment) OR (classroom physic* learning environment) OR (classroom soci* learning environment) were used in both CSSCI and SSCI databases. In the CSSCI database, these terms are translated into Chinese. In this study, the “back-translation” procedure proposed by Brislin (1970) was used in processing CSSCI search terms, i.e., the search terms were first translated from English to Chinese and then back-translated into English by a bilingual English Chinese speaker to ensure the quality of the translation and to obtain equivalent meanings.

As for the time span, we examined articles published between 1969 and 2022 in the SSCI sample, and for the CSSCI sample, we looked at the period from 2008 to 2022. This temporal scope was chosen to provide us with an extensive overview of the development and trends in the field over time.

The type of literature we included were peer-reviewed articles and provided rigorous insight into the topic at hand. Additionally, given the linguistic conventions of the databases, we included articles written in English for the SSCI database and articles written in Chinese for the CSSCI database.

Applying these criteria, we collected a total of 3,139 sources from the SSCI database and 1,261 sources from the CSSCI database. These formed the basis of our study and the ensuing analysis.

### The rationale for the inclusion criteria

Regarding the first criterion, The Classroom Learning Environment (CLE) is fundamentally characterized by four interrelated components—psychological, sociological, pedagogical, and physical—as identified by several authors (e.g., Fraser, 1998, 2011; Fan and Dong, 2005; Cai et al., 2022a, b). These four components were selected because they encompass the multi-dimensionality and complexity of the learning environment, contributing to a thorough and integrative perspective.

The psychological component involves the character attributes, mental health, and the general psychological atmosphere that both teachers and students bring into the learning environment (Fraser, 2001). The sociological aspect emphasizes the interaction dynamics in the classroom, which includes the relationships between teachers and students and interactions among peers (Cai et al., 2022b). Understanding these two critical components of the CLE can empower educators with tactics for implementing classroom interventions aimed at fostering student motivation and self-regulation. This is corroborated by the study of Cai et al. (2022b), which affirms the importance of psychosocial factors in boosting students’ perceived task value and self-regulation.

The pedagogical dimension evaluates the instructional methods and assessment techniques used (Cai et al., 2022a). Understanding teaching methods and assessments can promote student motivation and self-efficacy in completing the task (Leenknecht et al., 2020; Sapan and Mede, 2022).

The physical facet relates to the availability and use of teaching materials and the environmental conditions that can either foster or hinder effective learning (Fan and Dong, 2005). This perspective is further supported by the research conducted by Barrett et al. (2015), which highlights the significant role that physical setting plays in students’ academic progress.

Regarding the second criterion, WoS was selected because it is an extensive, comprehensive, and multidisciplinary database. It contains not only the metadata of the citing literature, such as title, author, institution, country, keywords, etc. but also the cited references in the paper. Most importantly, WoS naturally includes articles published in the Social Science Citation Index (SSCI) journals analyzed in this paper (Wang and Lv, 2021) as Gong and Cheng (2022) suggest that when it comes to international publications in social sciences, bibliometricians usually use SSCI. In addition, CSSCI-indexed journals provide China’s highest level and most innovative research (Shi and Li, 2019). Most importantly, the CSSCI database provides data for literature citation analysis, which is precisely what we need to investigate the knowledge base of CLE in China. Therefore, we included international publications from the SSCI database and Chinese publications from the CSSCI database.

Regarding the third criterion, the purpose of this article was to examine CLE from its inception to its development, so we did not limit the study’s time frame.

Regarding the fourth criterion, Wang and Lv (2021) pointed out that articles undergo rigorous peer review and represent original research, thus mirroring the interests of a particular field. Therefore, we included articles in our data collection.

Regarding the fifth criterion, the primary language of international articles is English, while the dominant language of Chinese research articles is Chinese. Therefore, we selected papers in English in SSCI and Chinese in CSSCI.

### Data analysis

We divided the data analysis into two parts. First, Excel software created a statistical map of the annual distribution of literature publications on CLE. Second, this study conducted a bibliometric analysis using CiteSpace 6.1.R4 and CiteSpace 6.1.R6 visualization software.

In addressing our research questions, the study conducted a combination of five techniques of analysis: collaborative network analysis, keyword co-occurrence analysis, keyword cluster analysis, burst analysis, and co-citation analysis. This combined approach allowed us to address the characteristics, hotspots, cutting-edge, and foundation knowledge of CLE research (Zhong et al., 2008; Guo, 2021). Most importantly, combining these five analyses enabled us to identify potential research gaps in CLE for future investigation.

### Collaborative network analysis

Network density reflects the degree of overall cooperation between research forces. In the CiteSpace software settings interface, this study set the network node type to “Author” and “Country” separately for author and country network analysis. “Author” is the joint author analysis function, which is helpful to reveal the leading author group and collaborative relationships in the classroom learning environment. “Country” is a country collaboration analysis function that allows you to show the distribution of collaboration fields and the intensity of collaboration between different countries.

### Keyword co-occurrence analysis

Keyword co-occurrence mapping enables researchers to examine the prominent themes and their development over time within a field. Using the CiteSpace software, the node type was set to “Keyword,” the time slice was set to “1,” and the threshold was set to Top 50, meaning the 50 most frequently cited keywords per year were chosen as the segmentation point for creating a keyword co-occurrence map for CLE research from data samples.

### Keyword cluster analysis

We carried out a keyword cluster analysis using CiteSpace software to identify potential common themes in CLE research. This was achieved by applying the log-likelihood ratio (LLR) algorithm which is designed to extract significant noun phrases from the titles keywords and abstracts of publications. These highly ranked noun phrases are then employed as labels for the respective clusters (Ekanayake et al., 2019).

### Burst analysis

**Figure 1:**
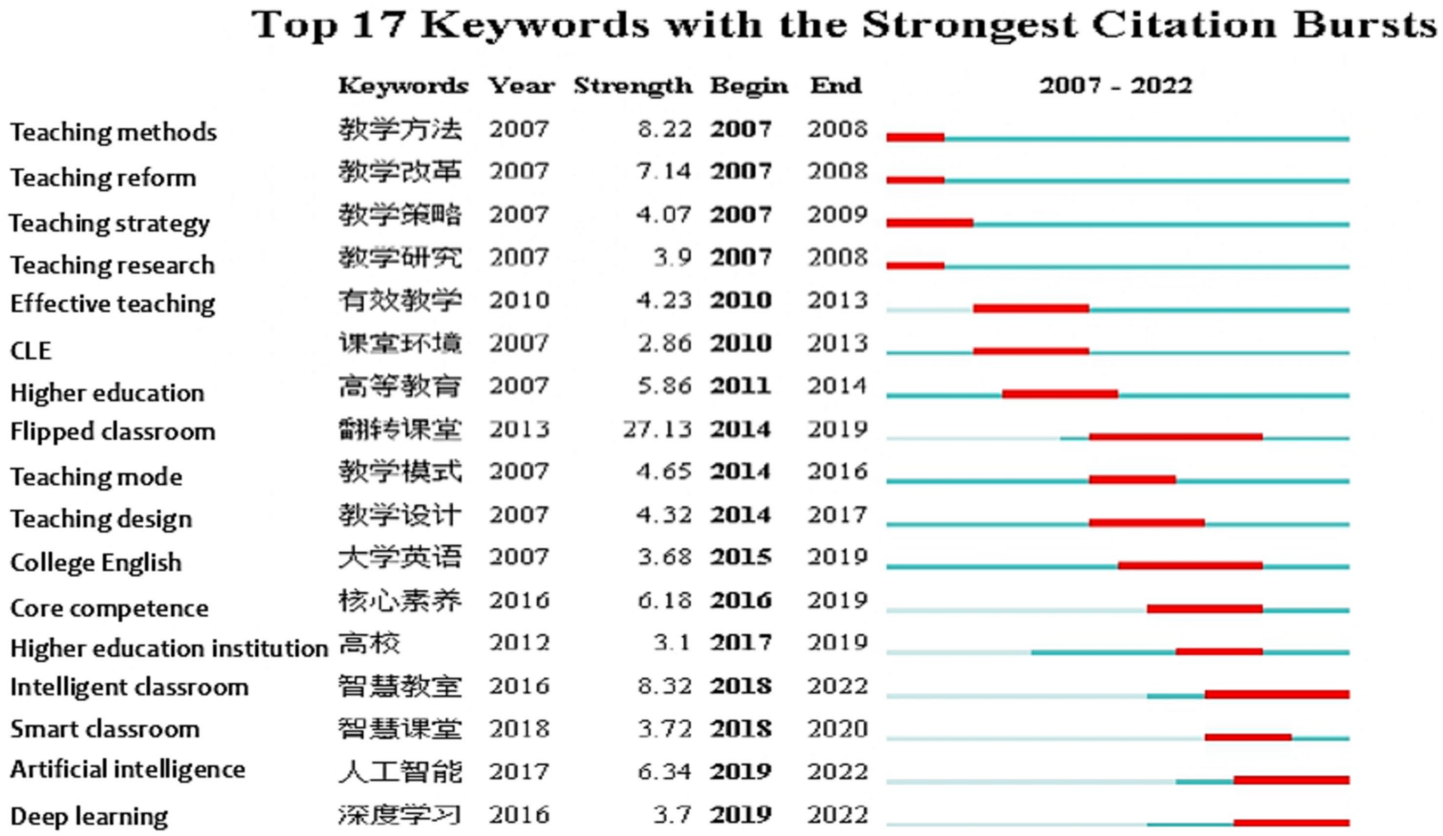
The citation burst of keywords in CLE from CSSCI.

**Figure 2:**
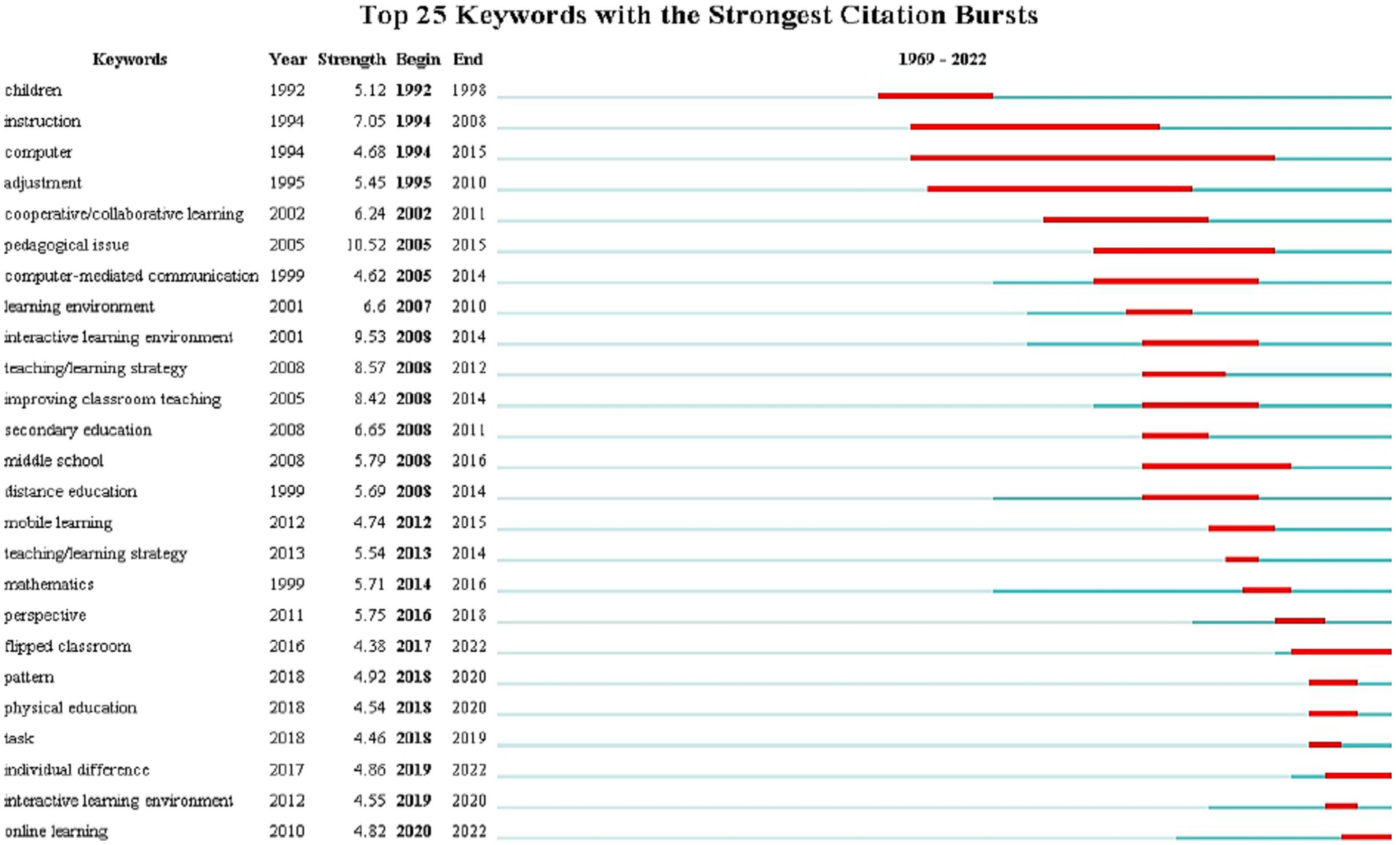
The citation burst of keywords in CLE from SSCI.

Burst analysis can help researchers explore the most advanced, latest, and promising research topics in a field, namely cutting-edge, represented by the sudden appearance of burst terms over a while. In this paper, all keywords in the literature were detected using the “Burstness” option in CiteSpace software, and the results are shown in Figures 1, 2. “The higher the burst rate of a term, the greater the contribution of the term to the frontier of the field. The year of the end of burst indicates the most recent progress in a research field, with the more recent year of the end of burst indicating the most recent trends in the field” (Guo, 2021, p. 33).

### Co-citation analysis

In the CiteSpace software settings interface, the node type was set to “Reference,” and the slicing method was selected as “Pathfinder” to conduct the co-citation analysis to identify the most frequently cited literature on CLE in China and abroad (see Tables 1, 2), which forms the knowledge base for CLE.

**Table 1:** Top 10 keywords in the field of CLE research from CSSCI and SSCI.

Top 10 keywords in CSSCI				Top 10 keywords in SSCI			
Rank	Frequency	Centrality	Keyword	Rank	Frequency	Centrality	Keyword
1	407	0.48	Teaching in CLE 课堂教学	1	458	0.15	Classroom
2	109	0.14	Flipped classroom 翻转课堂	2	358	0.08	Education
3	84	0.5	Teaching models 教学模式	3	354	0.01	Student
4	42	0.32	Teaching design 教学设计	4	246	0.08	Achievement
5	33	0.07	University students 大学生	5	224	0.06	Environment
6	31	0.11	Information technology 信息技术	6	196	0.14	Performance
7	27	0.06	Teaching quality 教学质量	7	193	0.05	Teacher
8	24	0.18	Classroom learning environment 课堂环境	8	187	0.07	Science
9	24	0.23	College English 大学英语	9	186	0.04	Perception
10	21	0.04	Teaching methods 教学方法	10	168	0.17	Children

**Table 2:** Hot topics in classroom learning environments.

Hot topics in CSSCI	Keywords	Hot topics in SSCI	Keywords
Pedagogical aspects	Teaching models, teaching design, teaching reform, teaching quality, and teaching methods	Science education	Science, mathematics, science education, STEAM education
Information technology	The flipped classroom, information technology, smart classroom, classroom video interaction, MOOC	Distance education	Computer-mediated communication, information technology, online learning, and blended learning
English teaching	College English, medical English, university student, English curriculum, English literature	Psychosocial CLE on learning outcomes	Student achievement and performance
The Influence of English CLE on students’ cognitive and affective outcomes	Students’ academic achievement or performance, learning attitudes, learning behaviors, and academic emotions	Student’s perception	Student, children, and student perceptions

The settings in CiteSpace were configured as follows: (1) Time slices spanning from 1969 to 2022 for the SSCI sample and from 2008 to 2022 for the CSSCI sample, with 1 year per slice. (2) Node types included country, author, and keyword. (3) For collaboration network and co-occurring network analysis, the top 50 most cited or most frequently occurring items were selected from each slice for countries, authors, and keywords, as recommended by Chen (2006). By setting a threshold for the top 50 keywords, we were able to focus on the most influential or representative entities within the research domain, therefore providing a clearer and more concise overview of the field. (4) Pruning was set to pathfinder and pruning of the merged network. To achieve the most prominent network, a pathfinder was used to remove redundant or counterintuitive connections (Song et al., 2016; Huang et al., 2020). All other settings were left at their default values.

## Results

### Overall development of CLE

**Figure 3:**
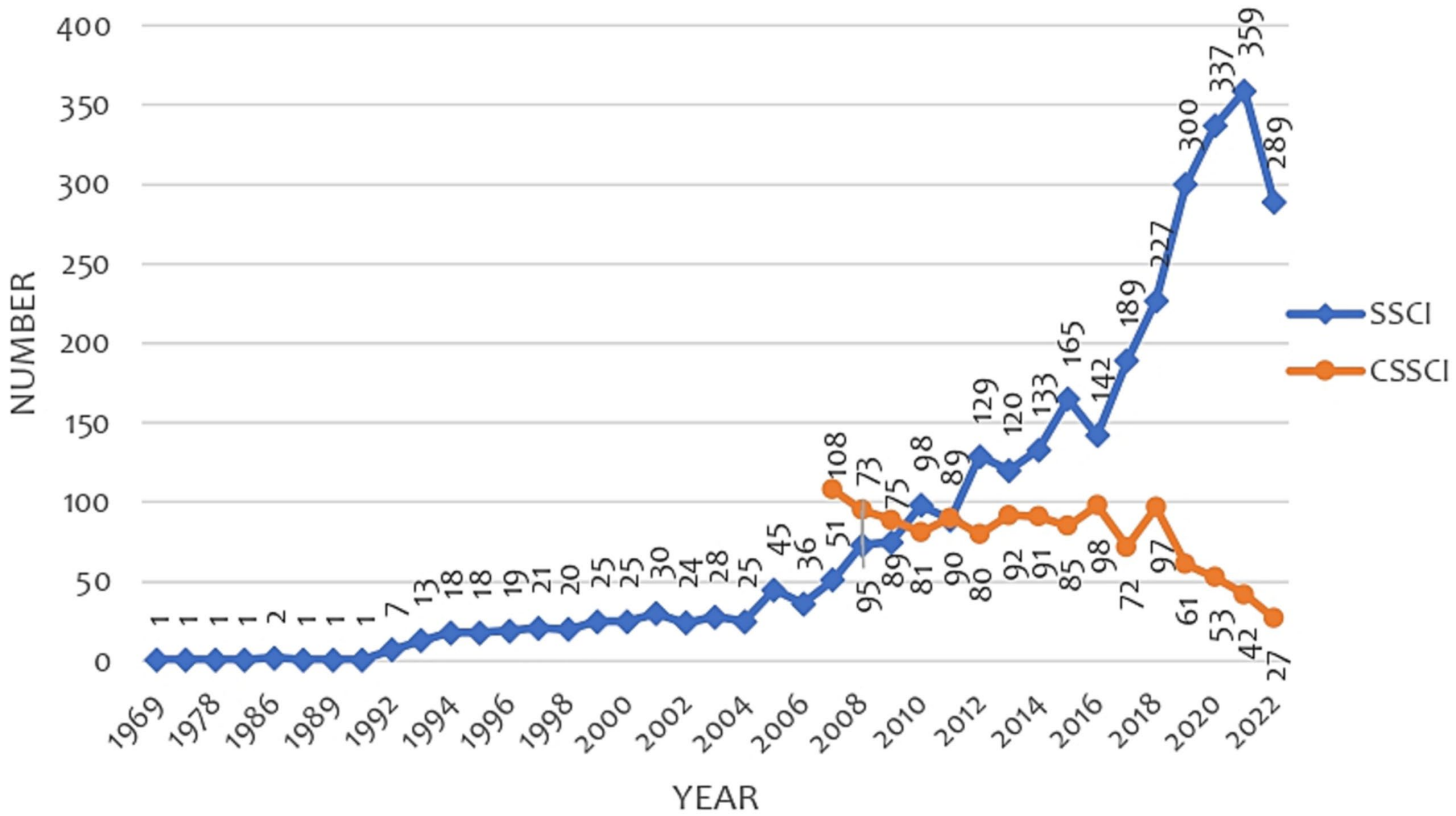
The academic output in the classroom learning environment.

The data analysis in Figure 3 shows that the number of English-language articles on CLE research is steadily increasing. In 2021, the number of published articles peaked at 359. In contrast, the number of Chinese articles on CLE research is steadily decreasing. In 2008, the number of published articles peaked at 108.

Table 3 reveals the leading 10 countries in terms of the number of publications related to CLE. China and the United States stand out with more than 1,000 articles each, with China contributing 1,701 and the United States 1,254. This volume of research surpasses any other country significantly. Australia and England followed with a significant number of articles, 230 and 200, respectively.

**Table 3:** Top 10 countries for publications of CLE.

Rank	Country	Number of publications	Centrality	Proportions (%)
1	CHINA	1701	0.58	39%
2	US	1,254	0.43	29%
3	AUSTRALIA	230	0.18	5%
4	ENGLAND	200	0.27	5%
5	CANADA	144	0.12	3%
6	SPAIN	121	0.06	3%
7	TURKEY	115	0.08	3%
8	GERMANY	94	0.09	2%
9	FINLAND	60	0.05	1%
10	NETHERLANDS	59	0.04	1%

Furthermore, the centrality values for China (0.58), the United States (0.43), England (0.27), and Australia (0.18) are high. This suggests that these countries occupy a crucial mediating position in CLE research.

### Author network analysis

Regarding the CSSCI sample, Liu Qingtang ranks first (see Table 4) in the Chinese sample regarding the number of published articles, with five articles mainly analyzing the impact and application of artificial intelligence (A.I.) on classroom teaching behavior. For example, Liu et al. (2019) constructed an intelligent analysis model for classroom teaching behavior based on artificial intelligence, which enables the analysis of classroom teaching behavior to be automated, normalized, and extended. Other authors with more publications include Li Rumi and Ding Rui, who mainly focused on teaching strategies and analyzed the relationship between CLE and students’ academic achievement or behavior (e.g., Ding et al., 2009). According to Price’s law formula, M ≈ 0.749 × √Nmax, Nmax is the number of papers by the author who contributed the most. In this study, Nmax was 5, and we calculated M as 1.7. A value greater than M means that the author is the primary author in the field. A leading author group is formed in a field when the total number of articles published by the primary author reaches 50% of all articles on the same topic (Price, 1963; Sun and Xiao, 2021). Therefore, we can conclude that the most productive authors in the field of CLE research have published more than two articles. In the Chinese sample, 21 authors have published more than two CLE articles, totaling 253, accounting for 20% of the total and less than 50% of the total, indicating that CLE research has not yet developed a stable core group of authors.

**Table 4:** Top 10 authors in CSSCI and SSC.

Top 10 authors in CSSCI			Top 10 authors in SSCI		
Rank	Author	Count	Rank	Author	Count
1	刘清堂 (Liu Qingtang)	5	1	Fraser, BJ	13
2	李如密 (Li Rumi)	5	2	Roth, WM	8
3	丁锐 (Ding Rui)	4	3	Yang, Harrison Hao	7
4	郑长龙 (Zheng Changlong)	4	3	Hwang, Gwo-Jen	7
5	何文涛 (He Wentao)	4	4	Nussbaum, Miguel	6
6	于素梅 (Yu Sumei)	4	4	Lee, Ju Seong	6
7	任庆梅 (Ren Qingmei)	4	5	Macleod, Jason	5
8	田爱丽 (Tian Aili)	4	5	Chen, Nian-Shing	5
9	汪颖 (Wang Yin)	3	6	Jegede, O.J.	4
10	刘秀梅 (Liu Xiumei)	3	6	Chen, Ying-Chih	4

**Figure 4:**
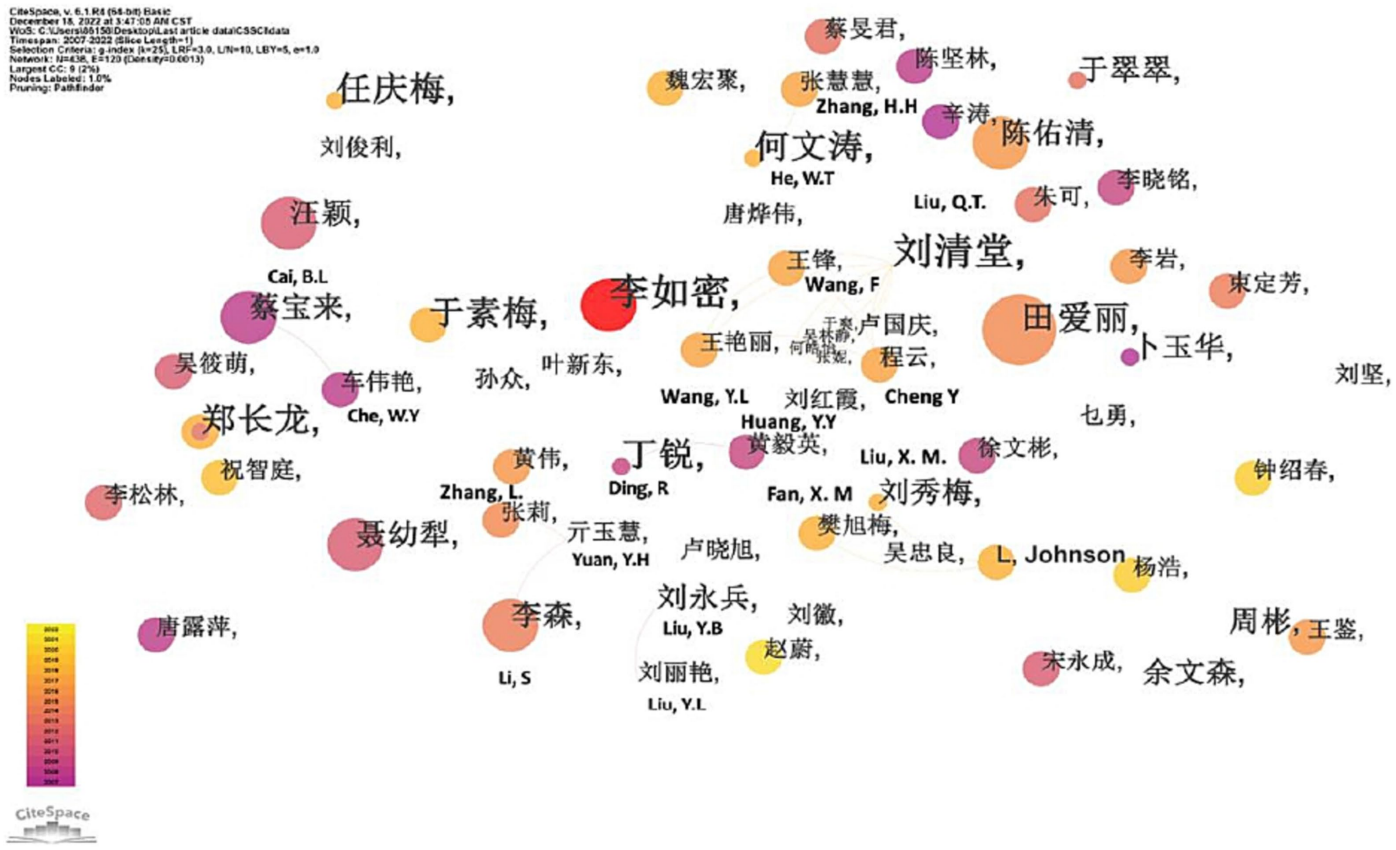
Visualization of co-author collaboration in CSSCI sample.

Figure 4 illustrates the formation of certain collaborative groups (CGs) among the key authors in this field. These include the following groups: Liu, Q.T., Wang, F., Cheng Y., Wang, Y.L. (i.e., CG1); Liu, X. M., Johnson, L., Fan, X.M. (i.e., CG2); Li, S., Zhang, L., Yuan, Y.H. (i.e., CG3); Ding, R. and Huang, Y.Y. (i.e., CG4); Cai, B.L. and Che, W.Y. (i.e., CG5); Liu, Y.L. and Liu, Y.B. (i.e., CG6); and lastly, Zhang, H.H. and He, W.T. (i.e., CG7).

Regarding the SSCI sample, Fraser ranks first in the English sample regarding the number of articles published, with 13 (See Table 4). The most important research papers deal with cross-cultural comparative studies on CLE. However, most of these studies are limited to validating CLE scales and analyzing participants’ perceptions of CLE from psychological and social perspectives. The second most frequent author is Roth, with eight articles whose research focuses on teacher-student interaction and student problem-solving in science classrooms. The other authors with more than five articles are Yang, Hwang, Nussbaum, and Lee. Again, based on Price’s Law formula, we calculated a threshold of 2.7 for prolific authors in the English sample. The most productive authors in CLE research were those who published more than three articles. In the English sample, 21 researchers published more than three articles on CLE, for a total of 102 pieces, representing 3.2% of the total, or less than 50%, suggesting that a stable core author group has yet to form in CLE research either.

**Figure 5:**
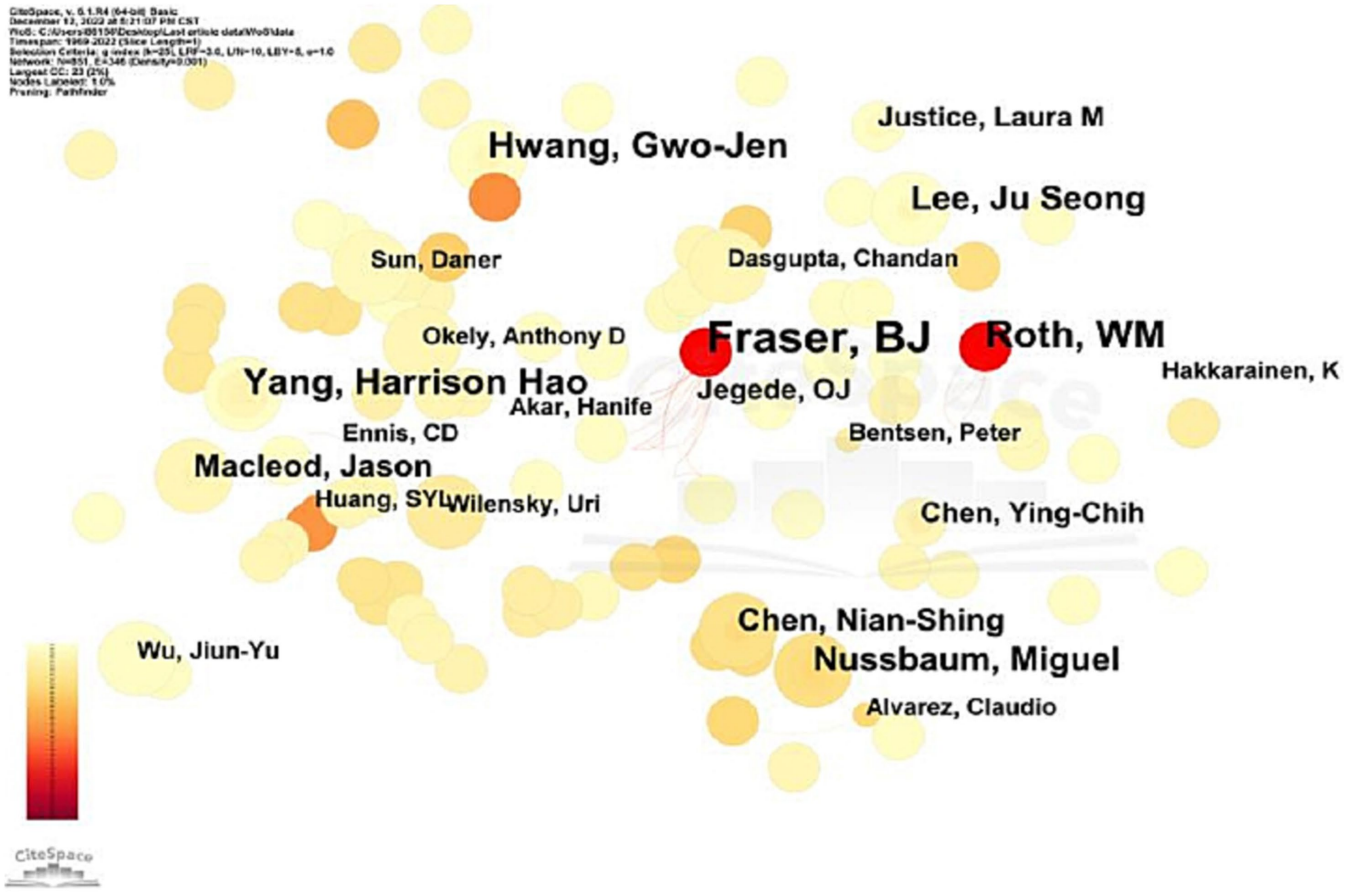
Visualization of co-author collaboration in SSCI sample.

As can be seen from Figure 5, certain collaborative group (CG) relationships were formed among the core authors: Fraser, B.J., Jegede, O. J and etc. (CG1); Roth, W. M, Bentsen, P and etc. (CG2); Nussbaum, M., Echeverria, A., Kong, S.C., Yin. C and etc. (CG3).

### Keyword co-occurrence mapping and keyword cluster analysis

Research hotspots represent the focal points of investigation within a particular field. Analyzing keyword co-occurrence maps can help researchers identify these hotspots. In our study, we conducted a keyword co-occurrence analysis using CSSCI and SSCI data.

**Figure 6:**
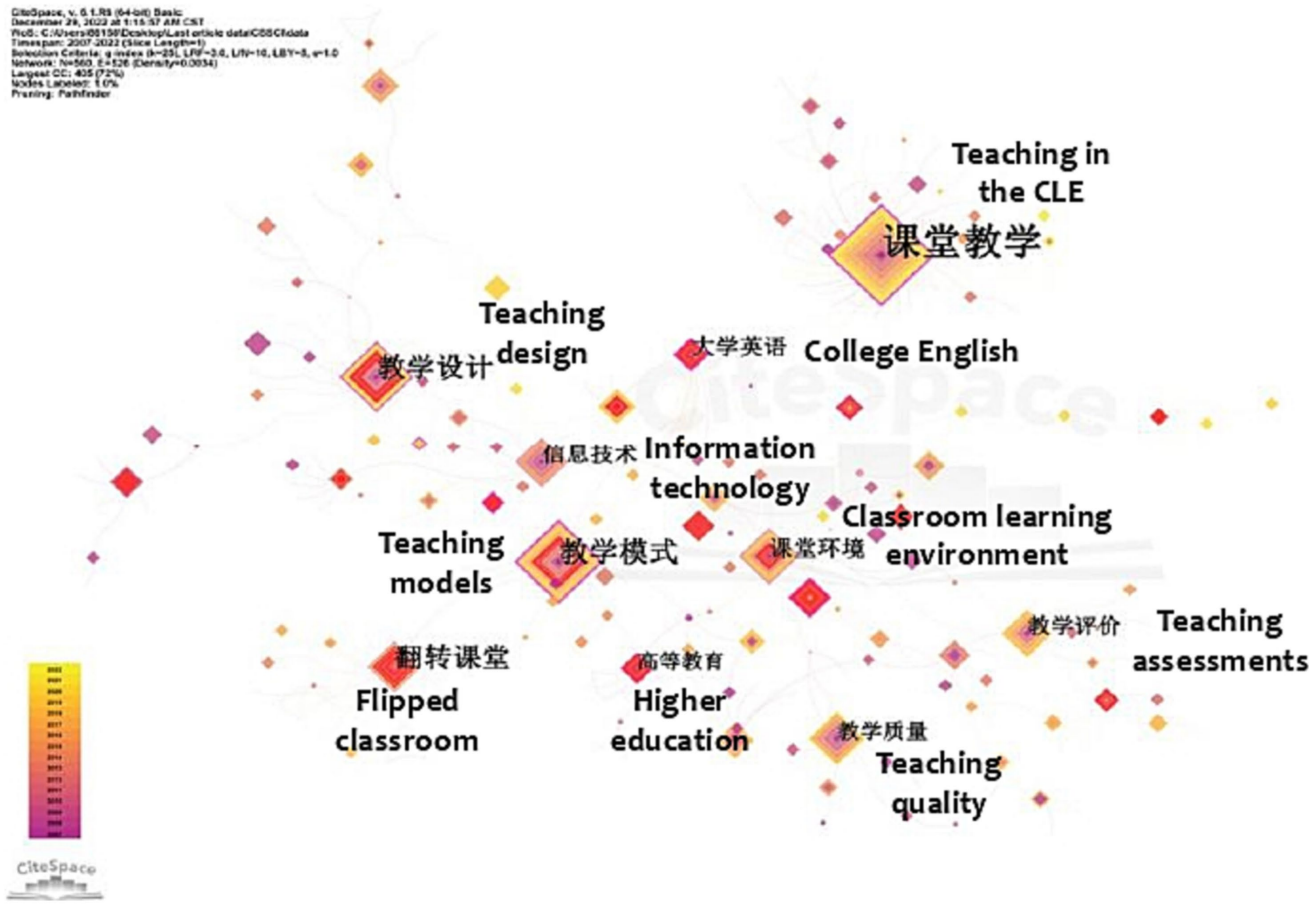
Keyword co-occurrence visualization network in the field of CLE from CSSCI.

In the CSSCI sample, Figure 6 displays 560 nodes and 526 lines. Larger diamond nodes indicate higher keyword frequency, while a darker outer color signifies more recent keyword co-occurrence. In Figure 6, the nodes representing keywords such as teaching in CLE, flipped classroom, teaching models, teaching design, teaching quality, and classroom learning environment have larger areas, suggesting their critical bridging role within the network structure. Frequently appearing keywords like university students, information technology, and college English are likely to emerge as prominent research topics (see Table 1).

**Figure 7:**
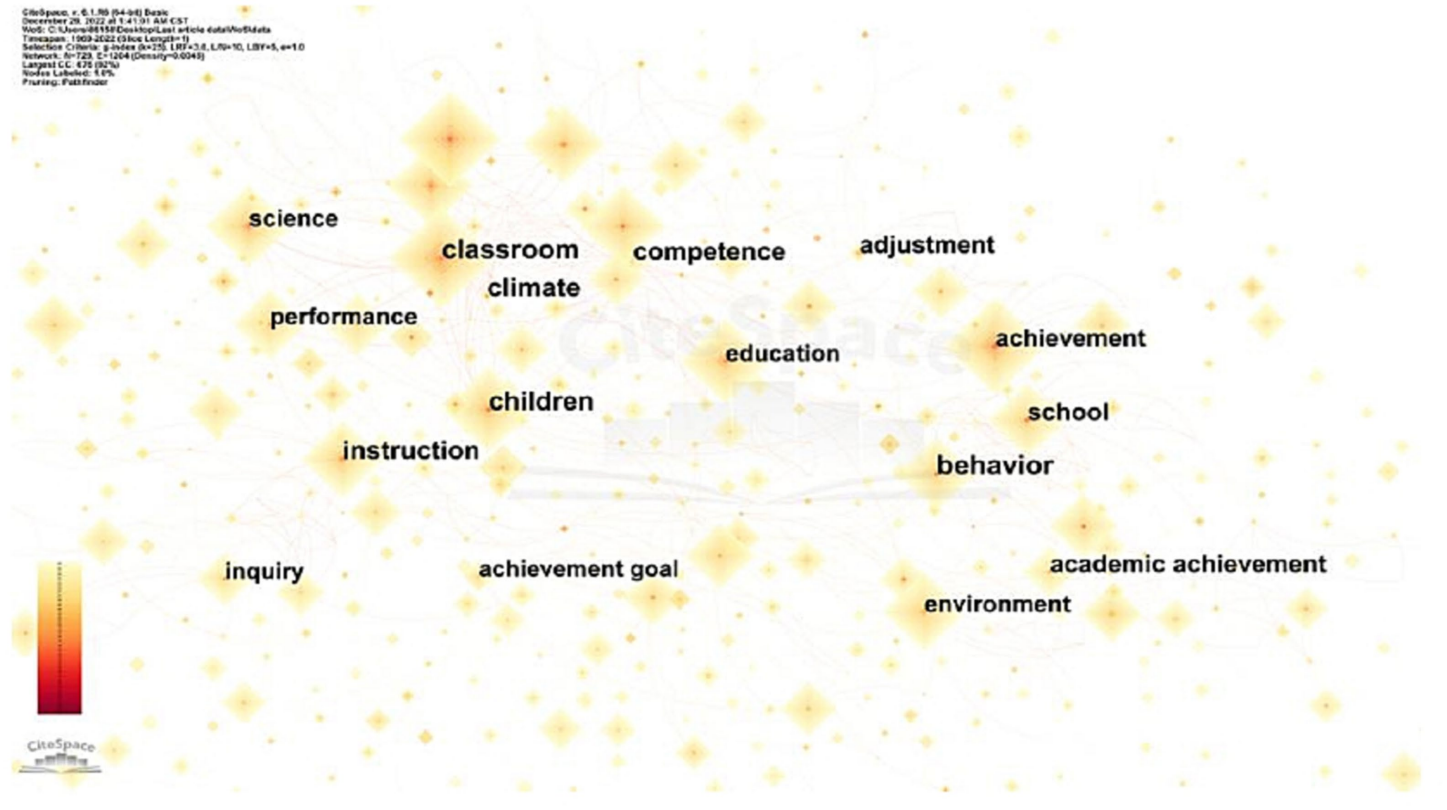
Keyword co-occurrence visualization network in the field of CLE from SSCI.

Likewise, in the SSCI sample, keywords such as classroom, education, and student held the most significant weight. The term environment was also frequently observed. Other keywords, like achievement, performance, teacher, science, perception, and children, have gained considerable attention as well (see Figure 7 and Table 1).

Subsequently, we further explored the analysis of CLE hotspots. To discover commonalities, we performed a keyword cluster analysis using the log-likelihood ratio (LLR) algorithm based on Keyword co-occurrence mapping analysis. Keyword clustering analysis focuses on the frequency of co-occurrence as the subject of examination. It employs statistical clustering statistical techniques to condense intricate co-occurrence network connections into relationships among a relatively smaller set of groups (Zhong et al., 2008).

Through keyword cluster analysis, we discerned seven clusters in the CSSCI sample and eleven clusters in the SSCI sample. Extracting precise information from a single cluster name poses a challenge and calls for an extensive analysis, paired with the main keywords within the cluster (Huang et al., 2020).

Therefore, we conducted an in-depth examination of the primary keywords within the clusters and performed extensive research on the abstracts or scholarly works in which the highest-ranked keywords were cited. This process encompassed a review of keyword co-occurrence analysis, and how the data were collected and categorized. Consequently, we established four principal categories of research hotspots, represented succinctly in both the CSSCI and SSCI samples, as illustrated in Table 2.

### CLE research hotspots in CSSCI samples

“Teaching models,” “Teaching design,” “Teaching reform,” “Teaching quality,” and “Teaching methods” appear among the top ten terms. Thus, the first research focus in the CSSCI sample is the study of pedagogical aspects in CLE.

Teaching models are the stable structure of the teaching process in a certain environment under the guidance of instructional objectives, learning activities, and assessment (He, 1997). Models of CLE have a multi-contextual and multidisciplinary character because developments in science, technology, and social change have prompted researchers in the humanities and social sciences to study classroom teaching models and learn from different aspects and levels of other disciplines (Wang T. et al., 2018). Multidisciplinary research on instructional models focuses on task types of instructional models for physics, English, chemistry, and Chinese. Inquiry-based instructional models (Yang, 2007), teaching models in the context of deep learning, instructional models from a multimodal perspective, and models of interaction between English language instruction and instruction in microblogging are some of the current topics in the field of instructional models.

Teaching design refers to the process by which educators design the content, format, teaching methods, and tools of the curriculum following educational objectives and educational requirements (Zhang, 2000). Teaching design is beginning to be based on the realistic developmental level of students, emphasizing the natural and dynamic nature of the learning process and the contextual nature of the learning content (Wang et al., 2014).

Teaching quality is the extent to which educational objectives are achieved in the teaching and learning process (Qian and Zhao, 2023). Strategies for improving instructional quality and systems for evaluating instructional quality are the focus of research on teaching quality (e.g., Liang et al., 2021).

Teaching reform refers to the educational system’s need to make ongoing adjustments concerning teaching content, methods, objectives, and evaluation to adapt to social, economic, and technological development changes to address students’ needs (Zheng, 2007; Bae, 2008). The focus of reform in classroom teaching revolves around: “learning needs to make a connection with students’ real life, students need to be more active in learning, teacher-student interaction should become effective, subject teaching should be integrated, teaching process should become dynamic, teaching content should be structured, teaching strategies should be integrated, teaching materials should become high-quality, teaching objectives should become individualized, and teaching evaluation should be diversified” (Zheng, 2007, p. 28). The overall reform of teaching is evolving toward diversified innovation and quality education.

The second research hot topic is integrating information technology (I.T.) into CLE, the keywords for which are “flipped classroom” and “information technology.” The impact of I.T. on classroom teaching and learning (Zhang, 2010) and the emergence of a new teaching method based on the developments of I.T., namely the “flipped classroom,” are the main research areas of the second research focus. Empirical studies on the flipped classroom model (Zhang, 2015) and the application of multidisciplinary flipped classrooms are the main streams of current research on the flipped classroom (e.g., Xing and Dong, 2015).

The third hot research topic is the environment of “English teaching” in universities. Tracing the 24-citing literature in which the keyword university English appears, it was found that research on the teaching environment in university English is mainly concerned with the mechanisms that influence the construction and evaluation of an effective teaching environment in university English. For example, Ren (2018) analyzed the construction and evaluation of the learning environment in university English classes based on four potential variables: learning behavior, interpersonal support, contextual support, and eagerness to learn.

The fourth research hot topic is the influence of English CLE on students’ cognitive and affective outcomes. According to Astin (2012) classification of students’ learning outcomes, the learning cognition mentioned in this article mainly refers to students’ academic achievement or performance (Zhu et al., 2019). The affective outcomes refer to students’ learning attitudes (Liu and Liu, 2012), learning behaviors (Zhao and Xu, 2012), and academic emotions (Xia and Xu, 2018).

### CLE research hotspots in SSCI samples

The first hot research topic is science education. After further reading the citing articles on science education, current science education research focuses mainly on the CLE social levels, such as teacher support, instructor relations, and student relations, and the influence of CLE in science education on students’ self-regulation and engagement. For example, Strati et al. (2017) found that instrumental teacher support positively influences students’ academic engagement in science class. In particular, research on STEM (science, technology, engineering, and mathematics) is widespread. Chase et al. (2019) found that teacher support, such as constructive prompts, was positively associated with student completion of invention tasks STEM.

The second hot research topic is distance education, where keywords such as computer-mediated communication, virtual, and blended learning frequently appear in the top 10 keywords of “education,” “classroom,” and “environment.” Computer-supported collaborative learning (CSCL) was regarded as one of the ways of computer-mediated communication (Weinberger et al., 2005) to keep social networks and to do problem-based projects (PBL) effectively (Donnelly, 2010). From the original computer-supported learning environment to today’s blended learning approach, i.e., a mix of offline and online, cutting-edge approaches to blended teaching and learning, namely flipped classroom pedagogy and the impact on student learning are a hot research topic in distance education (e.g., de Brito Lima et al., 2022; Youhasan et al., 2022).

The third hot research topic is the impact of psychosocial CLE on learning outcomes, including the keywords “achievement” and “performance.” Through further analysis of the citing literature on achievement and performance, the influence of psychosocial CLE on learning outcomes can be divided into two categories: the influence of psychosocial CLE on learning cognition (e.g., Huang et al., 2021) and emotional outcomes (e.g., Frenzel et al., 2007; Buckingham et al., 2023).

The fourth hot research topic is that the analysis of the CLE from the student’s perspective is much more than from the teacher’s perspective since the keywords’ student’ and “children” together occurred a total of 522 times and the keyword “teacher” only occurred 193 times in the citing literature.

### Cutting-edge research

Figures 1, 2 show the top keywords with the most robust citation bursts for CLE in the CSSCI and SSCI samples, respectively. “Smart classroom” and “artificial intelligence” in the CSSCI sample, “flipped classroom,” “individual differences,” and “online learning” in the SSCI sample are keywords with high burst rates and the most recent year of burst, representing the most recent research frontiers.

### Knowledge base

The five most frequently cited articles in the CSSCI and SSCI databases were further analyzed regarding methodology and research content.

Among the five most cited articles in the CSSCI database, the first three used literature analysis, and the other two used empirical research (see Table 5). Among them, the article by Zhong et al. (2013) on *instructional design based on the flipped classroom concept in the I.T. environment* has the highest cited frequency and has a co-citation relationship with several frequently cited articles, significantly influencing other CLE research literature. This article uses literature analysis to introduce the flipped classroom’s background, rationale, and current research status and constructs a Taiji loop model.

**Table 5:** Top 5 co-cited references of CLE studies from CSSCI.

Number	Frequency	Burst	Author	Year	Title
1	29	5.89	Zhong et al.	2013	instructional design based on the flipped classroom concept in the I.T. environment
2	28	7.68	Zhang et al.	2012	Study on the flipped classroom teaching model
3	14	4.25	He	2014	From the essence of “flipped classroom” to the future development of “flipped classroom” in China
4	9	2.94	Lu et al.	2013	Empirical evidence and reflection in the flipped classroom
5	8	3.28	Zhang et al.	2016	A study on the characteristics of interactive behavior in primary school mathematics classroom teaching in a smart classroom environment

Regarding research topics, the most cited literature in China focuses on flipped classroom research. Theoretically, the literature presents the flipped classroom development path and its challenges and difficulties (e.g., He, 2014). The role and effectiveness of flipped classrooms are reviewed at the practical level (e.g., Wang and Zhang, 2016).

The five most cited pieces of literature in the SSCI database use qualitative and quantitative research methods (see Table 6). Of these, the book *Qualitative data analysis: an expanded sourcebook* by Miles and Huberman (1994) has the highest cited frequency and had a greater impact on the literature for other studies on CLE from a qualitative methodological perspective.

**Table 6:** Top 5 co-cited references of CLE studies from SSC.

Number	Frequency	Burst	Author	Year	Title
1	46	5.51	Miles & Huberman	1994	Qualitative data analysis: an expanded sourcebook
2	33	10.78	Ryan & Deci	2017	Self-determination theory: basic psychological needs in motivation, development, and wellness
3	29	12.33	Freeman et al.	2014	Active learning increases student performance in science, engineering, and mathematics
4	25	6.83	Merriam	1998	Qualitative research and case study applications in education. Revised and expanded from “case study research in education.”
5	21	0	Gay	2018	Culturally responsive teaching: theory, research, and practice

In terms of research topics, the most cited English literature focuses on qualitative methodological research and case study validation (Miles and Huberman, 1994; Merriam, 1998), classroom teaching and learning (Freeman et al., 2014; Gay, 2018), and profiling self-determination theory (Ryan and Deci, 2017). In particular, Freeman et al. (2014) indicated that active learning might be a preferred and empirically validated instructional practice to improve student achievement in science, technology, engineering, and mathematics (STEM) subjects. Ryan and Deci (2017) analyzed six mini-theories of self-determination theory and the application and practice of self-determination theory in different domains and social contexts.

## Discussion

This study’s findings reveal that the number of CLE publications is growing in the SSCI sample while it is declining in the CSSCI sample. The countries with the highest productivity in this field are China, the United States, and Australia. Analysis of keyword occurrence and clusters indicates that the primary research topics in the CLE domain include pedagogical aspects, information technology, English teaching, and the impact of English CLE on students’ cognitive and emotional outcomes in the CSSCI sample, as well as science education, distance education, psychosocial CLE effects on learning outcomes, and student perceptions in the SSCI sample. Additionally, keyword burst analysis highlights that the emerging research trends or frontiers mainly focus on smart classrooms and artificial intelligence in the CSSCI sample, flipped classrooms, individual differences, and online learning in the SSCI sample.

### Comparison of the literature from CSSCI and SSCI

Regarding the distribution of countries in CLE research, China ranks first, indicating that Chinese researchers attach great importance to CLE research. Regarding the quantity and quality of CLE research, China has made significant contributions, consistent with Bai’s findings (Bai, 2022). After 2010, Chinese scholars contributed to studying the concept of CLE and research tools, which is more localized. However, Chinese research on CLE is gradually decreasing compared with other Western countries. One possible explanation for this trend could be the varying terminology used in China to describe CLE, such as learning settings and classroom climate or technology-centered CLE. This variation in nomenclature suggests that this study may not encompass all different CLE terminology in China.

When comparing author network analyses from Chinese and English literature, we noticed a higher degree of collaboration among Chinese authors. This is evidenced by the establishment of seven co-authorship clusters in China, juxtaposed with only three in international contexts. Moreover, in the Chinese context, 21 individual authors contributed more than two CLE articles, making up a total of 253 articles. This comprises 20% of the total output, yet falls short of the 50% mark. Similarly, in the English context, 21 individual researchers produced more than three CLE articles, collectively accounting for 102 articles, or 3.2% of the total, which is also less than half of the overall output. These patterns point to the absence of a consistent, core group of authors in both China and the West. In conclusion, these findings suggest the need for better information exchange and systematic collaboration among researchers, positioning these as future trends in CLE research.

Regarding hot research topics, the main similarities between Chinese and Western studies are first evident in the research on the cognitive and affective aspects of learning outcomes in CLE. Both Chinese and Western studies have investigated the effects of CLE on students’ academic scores. However, the difference is that Western studies have mainly analyzed the effects of CLE on student achievement from social and psychological aspects in science education. In contrast, Chinese studies have primarily focused on the pedagogical aspects of CLE in English language education. In addition, Western studies on the affective dimensions of learning outcomes addressed student motivation, engagement in learning, and self-regulation. In contrast, Chinese studies on the affective dimensions of learning outcomes have focused on learning attitudes, academic emotions, and classroom behaviors. Based on these discrepancies, we can conclude that the vast majority of CLE have focused only on the social, pedagogical, and psychological dimensions and that research on the physical dimensions of CLE or comprehensive analysis of CLE needs to be strengthened. This is consistent with the findings of Fan and Dong (2005).

This study shows that the current body of research on CLE has a primary focus on disciplinary subjects or single-subject studies. This observation is substantiated by the patterns that Chinese CLE research predominantly targets English language programs (e.g., Ren, 2018), while Western CLE studies are largely centered on science education programs (e.g., Strati et al., 2017). However, Wan and Cheng (2019) found differences in the effects of CLE on single-subject and interdisciplinary topics. Therefore, future researchers need to strengthen interdisciplinary research on CLE.

The main similarities between Chinese and Western studies are secondarily reflected in the research on applying information technology (I.T.) to CLE. One of the essential features is online learning, a pedagogy that uses electronic media and devices to support teaching and learning activities. Online learning can make learning and teaching more flexible and innovative through blended learning, digital or virtual technologies, and flipped classrooms (Tang et al., 2022). A flipped classroom is one in which students can watch an instructor-prepared learning video before class and focus on discussing problems with the instructor and peers during class (e.g., Chen et al., 2014). It is a shift from a teacher-centered approach to a student-centered learning model (Bhagat et al., 2016). This I.T. -supported learning model is at the cutting edge of Chinese and international CLE research. One difference, however, is that flipped classroom research in China has focused on multidisciplinary instructional practices and theoretical representations. In contrast, Western research has focused on testing the effectiveness of the flipped classroom, such as the impact of the flipped classroom model on student engagement and academic achievement.

Regarding the result of cutting-edge research, the smart classroom, in which advanced information technology and media devices are used to support digitally intelligent teaching and learning activities, is at the cutting edge of research on CLE in China (Chang and Lee, 2010), especially in the study of smart classroom construction and the characteristics of interactive teaching behaviors (e.g., Jin et al., 2018). This may be due to the release of the Education Informatics 2.0 Action Plan, which ushered in a new era of education informatics, in which strengthening the concept of competency-based talent education and building a new model of talent education under “Internet+” conditions became one of the fundamental goals of education informatics applications (Ministry of Education of the People's Republic of China, 2018). Building a smart classroom based on deep learning is the primary way to cultivate talents in the new era. Deep learning is learning based on understanding and pursuing the knowledge transfer application. The ultimate goal of a smart CLE is to promote deep learning (Yang and Wu, 2022).

In addition to the use mentioned above of I.T. in CLE, such as flipped classrooms and online learning, attention to individual differences is also at the cutting edge of research in Western CLE research. For example, gender differences in perceptions of CLE (e.g., Tas, 2016) and students’ different learning profiles, interests, and readiness have been studied in Western CLE research (e.g., Alwadei et al., 2020). This finding confirms that student differences are an essential component of the CLE and that ignoring these differences can affect student learning (Belfi et al., 2012). This diversity requires teachers to adopt instructional and assessment practices that adapt to individual learners’ different developmental needs and levels (UNESCO, 2017). Differentiated instruction and assessment are recommended in CLE research because differentiated instruction and assessment are effective in classrooms where both meet the diverse needs of students and have important implications for teachers (Tomlinson and Imbeau, 2010; Aldridge et al., 2012). Therefore, given that research on Chinese CLE has focused on the instruction rather than the students themselves, future research needs to develop and validate a new instrument that encompasses these two dimensions. In this regard, previous studies have yet to fully consider CLE teachers’ and leaders’ perspectives on CLE.

Although Chinese and Western CLE studies have used quantitative and qualitative analyses regarding the knowledge base outcome, existing studies have favored a single-report approach. Resonating with the suggestion by Ren et al. (2022), future studies might consider multiple-report measurements that combine self-and other-report assessments, which would facilitate testing the perceptions of different reporting individuals and exploring appropriate measurement approaches in other contexts.

In addition, most of the existing quantitative studies have used regression methods to examine the effects of CLE on student learning. However, these methods need to explain the internal mechanisms underlying the effects of CLE on student learning. Future research might consider examining the relationship between CLE and student learning using structural equation modeling (SEM) to create a system or model that includes mediating or moderating mechanisms. This would allow for a more reliable assessment of the internal relationships between variables (Jöreskog and Sörbom, 1998).

### Conclusions and limitations

Research has shown that the CLE can vary depending on cultural context (Aldridge and Fraser, 2000; Fraser et al., 2010). In particular, there are differences between Chinese and Western classroom learning environments (Lee et al., 2009). Therefore, studying the CLE across national borders is essential to gain insights into different educational systems and better understand the CLE in other countries (Fraser, 2023). To achieve this, this study conducted a bibliometric analysis and visualization to compare the English and Chinese CLE literature.

The most crucial theoretical contribution of comparing literature in English and Chinese on the CLE through bibliometric analysis is to provide a comprehensive understanding of the CLE field by identifying research gaps, visualizing research trends, and contributing to the development of a theoretical framework of CLE. For example, both Chinese and English literature on the CLE emphasize the impact of CLE on learning outcomes and technology integration. However, Chinese CLE focuses on English education and pedagogy, while English CLE research centers on science education and psychosocial aspects. These findings remind researchers to consider cultural factors when conducting CLE research and contribute to the development of a theoretical framework for CLE suitable for specific educational contexts by synthesizing research from different cultural contexts.

The practical contribution of comparing literature in English and Chinese on CLE through bibliometric analysis is to improve cross-cultural understanding and promote collaboration in research and practice regarding CLE.

Comparing literature in English and Chinese can provide policymakers and educators with a better understanding of how cultural differences affect CLE. For example, despite the Chinese national curriculum reform promoting student-centered teaching practices (Guo, 2013), teacher dominance is still prevalent in Chinese CLE because of historical and societal challenges (Lee et al., 2009). Chinese CLE places more emphasis on instruction than on the needs and preferences of individual learners. Therefore, teachers must adopt instructional and assessment practices that are adaptable to students’ diverse developmental needs and levels (UNESCO, 2017).

Although this study has comprehensively contributed to the cross-national understanding of CLE, there are some limitations to consider. First, even though previous studies have pointed out that an investigation may choose only one of the WoS and Scopus databases due to the possible overlap between these two databases (Wang and Lv, 2021), data from Scopus might also have provided an interesting perspective for this study. Second, other types of publications, such as reports and book chapters, may also be valid contributions to this research field, even if articles are strictly peer-reviewed and reflect the concerns of the field. Third, concerning the amount of literature considered and the inclusion criteria applied, although the current literature search effectively addressed our research questions at a broader level, future research could benefit from a more focused approach to delve deeper into the intricacies of CLE. A systematic review following the PRISMA (Preferred Reporting Items for Systematic Reviews and Meta-Analyses) guidelines could be useful (Morris et al., 2021) to extract the most relevant literature consistent with the specific research objectives.

## Data availability statement

The raw data supporting the conclusions of this article will be made available by the authors, without undue reservation.

## Author contributions

JC: conceptualization, data collection, and writing – original draft preparation. FD: writing – draft revising. GV: writing – review and editing. KL: supervision. All authors contributed to the article and approved the submitted version.

## Conflict of interest

The authors declare that the research was conducted in the absence of any commercial or financial relationships that could be construed as a potential conflict of interest.

## Publisher’s note

All claims expressed in this article are solely those of the authors and do not necessarily represent those of their affiliated organizations, or those of the publisher, the editors and the reviewers. Any product that may be evaluated in this article, or claim that may be made by its manufacturer, is not guaranteed or endorsed by the publisher.

## References

[ref1] Abacioglu C. S. Epskamp S. Fischer A. H. Volman M. (2023). Effects of multicultural education on student engagement in low-and high-concentration classrooms: the mediating role of student relationships. Learn. Environ. Res., 1–25. doi: 10.1007/s10984-023-09462-0

[ref2] Aldridge J. Fraser B. (2000). A cross-cultural study of classroom learning environments in Australia and Taiwan. Learn. Environ. Res. 3, 101–134. doi: 10.1023/A:1026599727439

[ref3] Aldridge J. M. Fraser B. J. Bell L. Dorman J. (2012). Using a new learning environment questionnaire for reflection in teacher action research. J. Sci. Teach. Educ. 23, 259–290. doi: 10.1007/s10972-012-9268-1

[ref4] Alwadei A. H. Tekian A. S. Brown B. P. Alwadei F. H. Park Y. S. Alwadei S. H.. (2020). Effectiveness of an adaptive eLearning intervention on dental students’ learning compared to traditional instruction. J. Dent. Educ. 84, 1294–1302. doi: 10.1002/jdd.12312, PMID: 32702776

[ref5] Astin A. W. (2012). Assessment for excellence: The philosophy and practice of assessment and evaluation in higher education. Lanham, Md. Rowman & Littlefield Publishers.

[ref6] Bae T. N. (2008). On the important transformation of the evaluation concept of classroom teaching quality in China. Educ. Res. 1:5.

[ref7] Bai Y. (2022). The composition and investigation of high school chemistry classroom environment. Master's thesis, Harbin Normal University.

[ref8] Barrett P. Davies F. Zhang Y. Barrett L. (2015). The impact of classroom design on pupils' learning: final results of holistic, multi-level analysis. Build. Environ. 89, 118–133. doi: 10.1016/j.buildenv.2015.02.013

[ref9] Belfi B. Goos M. De Fraine B. Van Damme J. (2012). The effect of class composition by gender and ability on secondary school students’ school well-being and academic self-concept: a literature review. Educ. Res. Rev. 7, 62–74. doi: 10.1016/j.edurev.2011.09.002

[ref10] Bhagat K. K. Chang C. N. Chang C. Y. (2016). The impact of the flipped classroom on mathematics concept learning in high school. J. Educ. Technol. Soc. 19, 134–142.

[ref11] Börner K. Chen C. Boyack K. W. (2003). Visualizing knowledge domains. Annu. Rev. Inf. Sci. Technol. 37, 179–255. doi: 10.1002/aris.1440370106

[ref12] Brislin R. W. (1970). Back-translation for cross-cultural research. J. Cross-Cult. Psychol. 1, 185–216. doi: 10.1177/135910457000100301

[ref13] Buckingham L. R. Fernandez Alvarez M. Halbach A. (2023). Differences between CLIL and non-CLIL students: motivation, autonomy, and identity. J. Multiling. Multicult. Dev. 44, 626–640. doi: 10.1080/01434632.2022.2102641

[ref14] Cai J. Wen Q. Jaime I. Cai L. Lombaerts K. (2022b). From classroom learning environments to self-regulation: the mediating role of task value. Stud. Educ. Eval. 72:101119. doi: 10.1016/j.stueduc.2021.101119

[ref15] Cai J. Wen Q. Lombaerts K. Jaime I. Cai L. (2022a). Assessing students’ perceptions about classroom learning environments: the new what is happening in this class (NWIHIC) instrument. Learn. Environ. Res. 25, 601–618. doi: 10.1007/s10984-021-09383-w

[ref16] Chang C. Y. Lee G. (2010). A major E-learning project to renovate science learning environment in Taiwan. Turkish Online J. Educ. Technol. 9, 7–12.

[ref17] Chase C. C. Marks J. Malkiewich L. J. Connolly H. (2019). How teacher talks guidance during invention activities shapes students' cognitive engagement and transfer. Int. J. STEM Educ. 6, 1–22. doi: 10.1186/s40594-019-0170-7

[ref18] Chen C. (2006). CiteSpace II: detecting and visualizing emerging trends and transient patterns in scientific literature. J. Am. Soc. Inf. Sci. Technol. 57, 359–377. doi: 10.1002/asi.20317

[ref19] Chen C. (2016). Cite space: a practical guide for mapping scientific literature (pp. 41–44). Hauppauge, NY, USA: Nova Science Publishers.

[ref20] Chen Y. X. Meng J. Yu H. B. (2017). A study of students' perceived classroom learning environment in higher education institutions. Educ. Dev. Res. 19, 54–60.

[ref21] Chen Y. Wang Y. Chen N. S. (2014). Is FLIP enough? Or should we use the FLIPPED model instead? Comput. Educ. 79, 16–27. doi: 10.1016/j.compedu.2014.07.004

[ref22] de Brito Lima F. Lautert S. L. Gomes A. S. (2022). Learner behaviors associated with uses of resources and learning pathways in blended learning scenarios. Comput. Educ. 191:104625. doi: 10.1016/j.compedu.2022.104625

[ref23] Ding R. Huang Y. Y. Lin Z. C. Ma Y. P. (2009). The relationship between classroom environment and learning outcomes in elementary school mathematics. Educ. Res. Exp., 73–80.

[ref24] Donnelly R. (2010). Interaction analysis in a “learning by doing” problem-based professional development context. Comput. Educ. 55, 1357–1366. doi: 10.1016/j.compedu.2010.06.010

[ref25] Dorman J. Adams J. (2004). Associations between students' perceptions of the classroom environment and academic efficacy in Australian and British secondary schools. Westminst. Stud. Educ. 27, 69–85. doi: 10.1080/0140672040270106

[ref26] Ekanayake E. Shen G. Kumaraswamy M. M. (2019). Mapping the knowledge domains of value management: a bibliometric approach. Eng. Constr. Archit. Manag. 26, 499–514. doi: 10.1108/ecam-06-2018-0252

[ref27] Fan C. L. Dong Q. (2005). Current status, implications, and trends in classroom environment research. Comp. Educ. Res. 26, 61–66.

[ref28] Fraser B. J. (1998). Classroom environment instruments: development, validity and applications. Learn. Environ. Res. 1, 7–34. doi: 10.1023/A:1009932514731

[ref29] Fraser B. J. (2001). Twenty thousand hours: editor's introduction. Learn. Environ. Res. 4, 1–5. doi: 10.1023/A:1011406709483

[ref30] Fraser B. J. (2011). Classroom environment (RLE Edu O). London, New York: Routledge.

[ref31] Fraser B. J. (2023). The evolution of the field of learning environments research. Educ. Sci. 13:257. doi: 10.3390/educsci13030257

[ref32] Fraser B. J. Aldridge J. M. Adolphe F. S. (2010). A cross-national study of secondary science classroom environments in Australia and Indonesia. Res. Sci. Educ. 40, 551–571. doi: 10.1007/s11165-009-9133-1

[ref33] Freeman S. Eddy S. L. McDonough M. Smith M. K. Okoroafor N. Jordt H.. (2014). Active learning increases student performance in science, engineering, and mathematics. Proc. Natl. Acad. Sci. 111, 8410–8415. doi: 10.1073/pnas.1319030111, PMID: 24821756 PMC4060654

[ref34] Frenzel A. C. Pekrun R. Goetz T. (2007). Perceived learning environment and students' emotional experiences: a multilevel analysis of mathematics classrooms. Learn. Instr. 17, 478–493. doi: 10.1016/j.learninstruc.2007.09.001

[ref35] Gay G. (2018). Culturally responsive teaching: theory, research, and practice. New York, NY: Teachers’ College Press.

[ref36] Gong K. Cheng Y. (2022). Patterns and impact of collaboration in China’s social sciences: cross-database comparisons between CSSCI and SSCI. Scientometrics 127, 5947–5964. doi: 10.1007/s11192-022-04483-7

[ref37] Guo L. (2013). New curriculum reform in China and its impact on teachers. Comp. Int. Educ. 41. doi: 10.5206/cie-eci.v41i2.9205

[ref38] Guo Z. (2021). International research on teacher burnout: knowledge base, hot topics, and frontier advance: a bibliometric analysis based on the WOS database Comparative Education Research.

[ref39] Guo M. Hu X. Leung F. K. (2022). Culture, goal orientations, and mathematics achievement among Chinese students. Int. J. Sci. Math. Educ. 20, 1225–1245. doi: 10.1007/s10763-021-10202-0

[ref40] He K. S. (1997). Constructivist teaching models, teaching methods and instructional design. J. Beijing Norm. Univ. Soc. Sci. Ed. 5, 74–81.

[ref41] He K. R. (2014). The future development of "flipped classroom" in China from the essence of "flipped classroom.". Res. Electro-Chem. Educ., 5–16. doi: 10.13811/j.cnki.eer.2014.07.001

[ref42] Huang B. Lu H. Zhu R. (2021). Disabled peers and student performance: quasi-experimental evidence from China. Econ. Educ. Rev. 82:102121. doi: 10.1016/j.econedurev.2021.102121

[ref43] Huang L. Zhou M. Lv J. Chen K. (2020). Trends in global research in forest carbon sequestration: a bibliometric analysis. J. Clean. Prod. 252:119908. doi: 10.1016/j.jclepro.2019.119908

[ref44] Jia Y. Way N. Ling G. Yoshikawa H. Chen X. Hughes D.. (2009). The influence of student perceptions of school climate on socioemotional and academic adjustment: a comparison of Chinese and American adolescents. Child Dev. 80, 1514–1530. doi: 10.1111/j.1467-8624.2009.01348.x, PMID: 19765015

[ref45] Jin X. Q. Tian X. S. Yang X. M. Du Y. (2018). Smart classroom construction and lesson analysis supported by big data. Mod. Educ. Technol. 28, 39–45.

[ref46] Jöreskog K. G. Sörbom D. (1998). LISREL 8: structural equation modeling with the SIMPLIS command language Scientific Software. Chicago: Scientific Software International, Inc.

[ref47] Lau K. L. Lee J. (2008). Examining Hong Kong students’ achievement goals and their relations with students’ perceived classroom environment and strategy use. Educ. Psychol. 28, 357–372. doi: 10.1080/01443410701612008

[ref48] Lee J. C.-K. Yin H. Zhang Z. (2009). Exploring the influence of the classroom environment on students’ motivation and self-regulated learning in Hong Kong. Asia Pac. Educ. Res. 18. doi: 10.3860/taper.v18i2.1324

[ref49] Leenknecht M. Wijnia L. Köhlen M. Fryer L. Rikers R. Loyens S. (2020). Formative assessment as practice: the role of students’ motivation. Assess. Eval. High. Educ. 46, 236–255. doi: 10.1080/02602938.2020.1765228

[ref50] Liang F. He W. Dong L. C. (2021). Strategies for improving the quality of mathematics classroom teaching in compulsory education in ethnic areas based on curriculum standards. Ethn. Educ. Res. 32, 107–114. doi: 10.15946/j.cnki.1001-7178.2021.06.013

[ref51] Liu Q. T. He H. Y. Wu L. J. Deng W. Chen Y. Wang Y.. (2019). Classroom teaching behavior analysis method based on artificial intelligence and its application. Chin. Electro-Chem. Educ., 13–21.

[ref52] Liu L. Y. Liu Y. B. (2012). A study on the relationship between English classroom environment and learning outcomes in high school. Theory Pract. Foreign Lang. Teach., 76–82.

[ref53] Merriam S. B. (1998). Qualitative research and case study applications in education. Revised and expanded from case study research in education. Jossey-bass Publishers, San Francisco, CA.

[ref54] Miles M. B. Huberman A. M. (1994). Qualitative data analysis: an expanded sourcebook. Thousand Oaks: Sage.

[ref55] Ministry of Education of the People's Republic of China. (2018). Education Informatization 2.0 Action Plan. Available at: http://www.moe.gov.cn/srcsite/A16/s3342/201804/t20180425_334188.html.

[ref56] Morris S. O’Reilly G. Nayyar J. (2021). Classroom-based peer interventions targeting autism ignorance, prejudice and/or discrimination: a systematic PRISMA review. Int. J. Incl. Educ., 1–45. doi: 10.1080/13603116.2021.1900421

[ref57] Ning B. Van Damme J. Van Den Noortgate W. Yang X. Gielen S. (2015). The influence of classroom disciplinary climate of schools on reading achievement: a cross-country comparative study. Sch. Eff. Sch. Improv. 26, 586–611. doi: 10.1080/09243453.2015.1025796

[ref58] Price D. J. (1963). Little science, big science. New York: Columbia University Press.

[ref59] Qian M. X. Zhao L. L. (2023). Big data enabled undergraduate teaching quality evaluation: value implications, practical dilemmas and path choices Chongqing Higher Education Research.

[ref60] Ren Q. M. (2018). The influence mechanism of constructing and evaluating effective classroom environment in college English. Foreign Lang. Teach. Res. 50, 703–714.

[ref61] Ren Y. C. Zhang J. W. Zhao F. (2022). A comparative study of team creativity in China and abroad since the 21st century. Res. Manag. 43:65.

[ref62] Rusticus S. A. Pashootan T. Mah A. (2023). What are the key elements of a positive learning environment? Perspectives from students and faculty. Learn. Environ. Res. 26, 161–175. doi: 10.1007/s10984-022-09410-4 35574193 PMC9076804

[ref63] Ryan R. M. Deci E. L. (2017). Self-determination theory: basic psychological needs in motivation, development, and wellness. New York: Guilford Publications.

[ref64] Sapan M. Mede E. (2022). The effects of differentiated instruction (DI) on achievement, motivation, and autonomy among English learners. Iran. J. Lang. Teach. Res. 10, 127–144. doi: 10.30466/IJLTR.2022.121125

[ref65] Shi J. M. Li J. (2019). Comparative analysis of domestic and foreign CSR research based on cite space. J. Chin. Acad. Soc. Sci. 65–73.

[ref66] Shu F. Ma Y. Qiu J. Larivière V. (2020). Classifications of science and their effects on bibliometric evaluations. Scientometrics 125, 2727–2744. doi: 10.1007/s11192-020-03701-4

[ref67] Song J. Zhang H. Dong W. (2016). A review of emerging trends in global PPP research: analysis and visualization. Scientometrics 107, 1111–1147. doi: 10.1007/s11192-016-1918-1

[ref68] Strati A. D. Schmidt J. A. Maier K. S. (2017). The perceived challenge, teacher support, and teacher obstruction as predictors of student engagement. J. Educ. Psychol. 109, 131–147. doi: 10.1037/edu0000108

[ref69] Sun Y. Xiao L. (2021). Research trends and hotspots of differentiated instruction over the past two decades (2000–2020): a bibliometric analysis. Educ. Stud., 1–17. doi: 10.1080/03055698.2021.1937945

[ref70] Tang Y. M. Lau Y. Y. Chau K. Y. (2022). Towards a sustainable online peer learning model based on student's perspectives. Educ. Inf. Technol. 27, 12449–12468. doi: 10.1007/s10639-022-11136-y PMC9157034 35668899

[ref71] Tas Y. (2016). The contribution of perceived classroom learning environment and motivation to student engagement in science. Eur. J. Psychol. Educ. 31, 557–577. doi: 10.1007/s10212-016-0303-z

[ref72] Tomlinson C. A. Imbeau M. B. (2010). Leading and managing a differentiated classroom (professional development). Alexandria, Va. 1st Edn ASCD.

[ref73] UNESCO. (2017). A guide for ensuring inclusion and equity in education. Paris: UNESCO.

[ref74] Velayutham S. Aldridge J. M. (2012). Influence of psychosocial classroom environment on students’ motivation and self-regulation in science learning: a structural equation modeling approach. Res. Sci. Educ. 43, 507–527. doi: 10.1007/s11165-011-9273-y

[ref75] Wan Z. H. Cheng M. H. M. (2019). Classroom learning environment, critical thinking and achievement in an interdisciplinary subject: a study of Hong Kong secondary school graduates. Educ. Stud. 45, 285–304. doi: 10.1080/03055698.2018.1446331

[ref76] Wang C. Boyanton D. Ross A.-S. M. Liu J. L. Sullivan K. Anh Do K. (2018). School climate, victimization, and mental health outcomes among elementary school students in China. Sch. Psychol. Int. 39, 587–605. doi: 10.1177/0143034318805517

[ref77] Wang J. Liu C. W. Chen W. D. (2014). Characteristics of future classroom instructional design: an embodied cognitive perspective. Mod. Distance Educ. Res. 71–78.

[ref78] Wang S. Lv X. (2021). Hot topics and evolution of frontier research in early education: a bibliometric mapping of the research literature (2001–2020). Sustainability 13:9216. doi: 10.3390/su13169216

[ref79] Wang T. Xu J. C. Wang A. J. (2018). Review and reflection on classroom teaching research in China in the past 40 years of reform and opening up. J. Educ. 27–34.

[ref80] Wang J. Y. Zhang Y. (2016). A study on the teaching effect of flipped classroom in secondary school physics based on modeling mechanism. Electrochem. Educ. Res. 37, 116–122. doi: 10.13811/j.cnki.eer.2016.09.018

[ref81] Weinberger A. Ertl B. Fischer F. Mandl H. (2005). Epistemic and social scripts in computer–supported collaborative learning. Instr. Sci. 33, 1–30. doi: 10.1007/s11251-004-2322-4

[ref82] Xia Y. Xu Y. (2018). A study on the influence of English major classroom environmental factors on students' negative academic emotions. Foreign language and foreign. Lang. Teach. 65–76. doi: 10.13458/j.cnki.flat.004493

[ref83] Xing L. Dong Z. H. (2015). A quasi-experimental study on the teaching effect of flipped classroom in university physics. Fudan Educ. Forum 1, 24–29. doi: 10.13397/j.cnki.fef.2015.01.006

[ref84] Yang F. Z. (2007). Exploration of the inquiry-based classroom teaching model. China University Teaching, 12, 6–12, 8.

[ref85] Yang C. Y. Wu F. T. (2022). A framework for smart classroom design based on deep learning. Open Educ. Res. 91–100. doi: 10.13966/j.cnki.kfjyyj.2022.06.010

[ref86] Youhasan P. Chen Y. Lyndon M. P. Henning M. A. (2022). University teachers' perceptions of readiness for flipped classroom pedagogy in undergraduate nursing education: a qualitative study. J. Prof. Nurs. 41:26. doi: 10.1016/j.profnurs.2022.04.001 35803656

[ref87] Zhang H. (2000). Instructional design research: a hundred-year review and a look ahead. Educ. Sci., 25–29.

[ref88] Zhang C. L. (2010). A pilot study on the impact of information technology on mathematics classroom teaching. Curriculum. Teaching materials. Teach. Methodol. 75–79. doi: 10.19877/j.cnki.kcjcjf.2010.01.016

[ref89] Zhang C. H. (2015). An empirical study on the factors influencing the acceptance of flipped classroom teaching model among full-time university teachers. J. South China Norm. Univ. (Soc. Sci. Ed.) 57–62.

[ref90] Zhao Q. H. Xu J. F. (2012). A study on the relationship between college English classroom environment and students' classroom behavior. Foreign language and foreign. Lang. Teach. 66–69. doi: 10.13458/j.cnki.flat.000529

[ref91] Zheng J. Z. (2007). Ten key points of classroom teaching change. Educ. Theory Pract. 6, 28–33.

[ref92] Zhong W. J. Li J. Yang X. J. (2008). The research of co-word analysis (3)-the principle and characteristics of the co-word cluster analysis. J. Inf. 7:118.

[ref93] Zhong X. Song S. Q. Jiao L. Z. (2013). A study of instructional design based on the concept of the flipped classroom in information-based environment. Open Educ. Res., 58–64. doi: 10.13966/j.cnki.kfjyyj.2013.01.003

[ref94] Zhu L. H. Zhang C. Y L. S. (2019). A study on college students' perceived classroom environment and learning style influence learning outcomes. J. Dalian Univ. Technol. (Soc. Sci. Ed.), 114–120. doi: 10.19525/j.issn1008-407x.2019.05.015

